# High Sensitive Detection of Carbohydrate Binding Proteins in an ELISA-Solid Phase Assay Based on Multivalent Glyconanoparticles

**DOI:** 10.1371/journal.pone.0073027

**Published:** 2013-08-27

**Authors:** Fabrizio Chiodo, Marco Marradi, Boris Tefsen, Harm Snippe, Irma van Die, Soledad Penadés

**Affiliations:** 1 Laboratory of GlycoNanotechnology, Biofunctional Nanomaterials Unit, CIC biomaGUNE, San Sebastián, Spain; 2 Networking Research Centre on Bioengineering, Biomaterials and Nanomedicine (CIBER-BBN), San Sebastián, Spain; 3 Department of Molecular Cell Biology and Immunology, VU University Medical Center, Amsterdam, The Netherlands; 4 Department of Medical Microbiology, University Medical Center Utrecht, Utrecht, The Netherlands; Duke University Medical Center, United States of America

## Abstract

Improved detection of anti-carbohydrate antibodies is a need in clinical identification of biomarkers for cancer cells or pathogens. Here, we report a new ELISA approach for the detection of specific immunoglobulins (IgGs) against carbohydrates. Two nanometer gold glyconanoparticles bearing oligosaccharide epitopes of HIV or *Streptococcus pneumoniae* were used as antigens to coat ELISA-plates. A ~3,000-fold improved detection of specific IgGs in mice immunized against *S. pneumoniae* respect to the well known BSA-glycoconjugate ELISA was achieved. Moreover, these multivalent glyconanoparticles have been employed in solid phase assays to detect the carbohydrate-dependent binding of human dendritic cells and the lectin DC-SIGN. Multivalent glyconanoparticles in ELISA provide a versatile, easy and highly sensitive method to detect and quantify the binding of glycan to proteins and to facilitate the identification of biomarkers.

## Introduction

The detection of anti-glycan antibodies in serum is of mounting interest for the evaluation of carbohydrate-based vaccines and pathogen infection as well as for the detection of biomarkers in diseases like cancer. The profiling of human serum antibodies has shown that a substantial part of circulating antibodies is directed against carbohydrates [1]. The affinity of anti-carbohydrate antibodies towards their epitopes, demands a multivalent presentation of the carbohydrate-ligands and highly sensitive screening methods. Furthermore, the low abundance of anti-carbohydrates antibodies in serum during pathological states and/or early infection hampers their use as biomarkers for prompt diagnosis. The coupling of carbohydrates on a scaffold (carrier) allows the multiple presentation of these antigens in an enzyme-linked immunosorbent assay (ELISA) [2]. However, while protein coating of ELISA plates is a well-established methodology, equivalent strategies for the direct coating of carbohydrates have been hampered by technical limitations. Early attempts to detect antibodies against bacterial polysaccharides by ELISA showed the difficulty to absorb carbohydrates to the supporting materials. This problem was solved by conjugation of the polysaccharides to positive charged poly-lysine scaffold, which allowed the immobilization of the resulting neoglycoconjugate to ELISA plates [3]. Soon afterwards, glycolipids were effectively employed to coat ELISA surfaces for a *Mycobacterium leprae*-specific serodiagnostic test [4–6]. With the identification and characterization of oligosaccharide antigen structures, chemical strategies were developed to conjugate the carbohydrate antigen to proteins (bovine or human serum albumin), polymers (acrylamide derivatives) [7,8] and dendrimers [9] in order to enhance the number carbohydrate epitopes on the ELISA surface and improve the sensitivity of the assay. The relatively recent introduction of glycan microarray technology also provided a platform for high throughput screening, yielding information about the specificity of glycan-binding proteins [10,11]. Although novel applications of glycan microarrays are emerging, including vaccine development and identification of disease specific biomarkers, ELISA is still the most widely used assay for diagnostic purposes. Its simplicity, sensitivity and low cost make ELISA a reliable competitor of newer screening methods for diagnosis such as printed glycan microarray technology. Although the comparison between glycan microarrays technology and conventional ELISA for evaluation of anti-carbohydrate antibodies led to contrasting opinions about sensitivity [9,12] the importance of both techniques as complementary screening methods has prompted the development of new immobilization strategies to improve their performance. For example, a new type of microarray based on silica glyconanoparticles has recently been described to study glycan-lectin interactions [13]. Here we describe the development of a fast and highly sensitive method, in which commercially available ELISA plates are directly coated with two nanometer gold glyconanoparticles (GNPs) [14] carrying carbohydrate antigens. We demonstrate that anti-carbohydrate antibodies can be detected in the nanomolar range by performing GNP-ELISA with the purified anti-HIV human monoclonal antibody 2G12 and with serum from mice immunized against *Streptococcus pneumoniae*. Moreover, we show that GNPs can be employed in a solid phase assay to profile the carbohydrate-binding of human cells and to profile lectins affinities on a highly multivalent surface. GNPs allow the introduction of a high number of carbohydrates on a nanometric gold scaffold [14] and the combination of different molecules on the same nanoparticle in a controlled way and with varying density [15,16]. We reasoned that the high number of carbohydrates on the GNPs (60-100 molecules) and their high molecular weight (around 50 kD) make GNPs very suitable for the ELISA plate coating in order to improve the sensitivity of the anti-carbohydrate antibody detection and study other carbohydrate-binding proteins.

## Materials and Methods

### Materials

All chemicals were purchased as reagent grade from Sigma-Aldrich, except chloroauric acid (Strem Chemicals), and were used without further purification. Anti HIV-1 gp120 Monoclonal Antibody 2G12 was kindly supplied by Dr D. Katinger (Polymun Scientific, Vienna, Austria).

### Preparation of GNPs

GNPs were prepared in a one-step reaction by reducing a gold salt with sodium borohydride in the presence of a mixture of thiol-functionalized glycoconjugates in the desired molar ratio following an established procedure [17]. GNPs bearing 10% of a thiol-ending conjugate of dimannoside Man(α1-2) Man(α1→) (DiMan), tetramannoside Man(α1-2) Man(α1-2) Man(α1-3) Man(α1→) (TetraMan) or Man(α1-2) Man(α1-3) [Man(α1-2) Man(α1-6)] Man(α1→) (PentaMan) and 90% of 5-(mercapto)pentyl β-D-glucopyranoside as inner component were prepared as previously reported [18]. GNPs carrying Gal(β1-4) Glc(β1-6) [Gal(β1-4)] GlcNAc(1→) (TetraPn) and OVA_323-339_ peptide were prepared following a protocol previously described [19]. The ratio of the different ligands on the nanocluster surface was determined by quantitative ^1^H NMR. Transmission electron microscopy showed an average gold core diameter of 2 nm.

### GNP-ELISAs

50 µL of a GNPs solution of 25µg/mL (or the reported concentration in the manuscript) in buffer (50mM Na _2_CO_3_, pH=9.7) were used to coat the Nunc MaxiSorp plate overnight at 4^°^C or 2h at room temperature. After discarding the GNPs solutions and washing with PBS (10mM, pH=7.4) (2x200µL), the wells were blocked with 200µL of 1% BSA (Sigma-Aldrich, lyophilized powder, ≥96%, agarose gel electrophoresis) in PBS at room temperature for 30 min. The blocking solution was discarded and 100µL of 2G12 (from 13.8 to 0.13nM) or 100µL of mice serum at different dilution in assay buffer (0.5% BSA) were added to the plate. After shaking for 1 h at 500rpm, the wells were washed with PBS (3x200µL) and then 100 µL of anti-human horseradish peroxidase (0.8 µg/mL, life technologies, Novex^®^ Goat anti-Human IgG-HRP) or 100 µL of anti-mouse horseradish peroxidase (0.8 µg/mL, life technologies, Novex^®^ Rabbit anti-Mouse IgG-HRP) were added for 2G12 or mice serum IgG detection, respectively. After 30 min of shaking at 500 rpm, the wells were washed with PBS (3x200µL). Finally, 100 µL of substrate solution (3,3′,5,5′-Tetramethylbenzidine, TMB, in citric/acetate buffer, pH=4, and H_2_O_2_) was added and after 3 min incubation at room temperature the reaction was stopped with 50 µL of H_2_SO_4_ (0.8 M) and the optical density was measured at 450 nm in an ELISA reader. These experiments were always performed in triplicate with independently prepared samples.

### In vitro generation and culture of human DCs

Immature DCs were generated from human peripheral blood mononuclear cells (PBMCs) as described previously [20] from buffy coats of healthy volunteers. The peripheral blood of healthy volunteers was used for isolation of PBMCs upon donor consent in accordance with the Declaration of Helsinki (Sanquin Blood bank, Amsterdam, The Netherlands). The Sanquin’s General Terms and Conditions can be founded following this link: http://www.sanquin.nl/repository/documenten/en/general-conditions/sanquin-blood-supply-general-terms-and-conditions-of-purchase-EN.pdf.

The Ethical Advisory Council of Sanquin can be checked following this link: http://www.sanquin.nl/en/about/about-sanquin/organisation/advisory-councils/ethical-advisory-council/.

Monocytes were prepared from PBMCs by centrifugation over Percoll and incubated for 5 days in RPMI supplemented with 10% heat inactivated fetal calf serum, 2.4 mM L-glutamine, 100 U/mL penicillin-streptomycin (all from Gibco), 800 U/mL of human recombinant granulocyte-macrophage colony-stimulating factor and 500 U/mL of human recombinant IL-4 (both from Schering-Plough, Brussels, Belgium).

### Cellular binding

Ninety-six-well plates (Nunc MaxiSorp^®^) were coated at room temperature for 2 h with 50 µL of GNPs (25 μg/mL) and afterwards blocked with 1% BSA. Calcein (calceine AM, Molecular Probes) was used to label moDC following a reported protocol [21]. Calcein labeled moDC were incubated on the GNPs-coated ELISA wells (40,000 cells/well) for 2 h at 37^°^C in calcium and magnesium containing TMS buffer (20 mM tris(hydroxymethyl)aminomethane (Tris)-HCl, pH 8.0; 150 mM NaCl; 1 mM CaCl_2_; 2 mM MgCl_2_) in presence or absence of 3.75 mM EGTA or 10 μg/mL of mAbs AZN-D1. Non adherent cells were then removed by gentle washing with 0.5% BSA-PBS solution and the adherent cells on the plate were lysed and the binding was correlated with the calcein absorption. The fluorescence was quantified on a Fluorostar spectrofluorimeter (BMG Labtech, Offenburg, Germany).

### DC-SIGN binding

Nunc MaxiSorp plates were coated with 50 μL GNPs (15 μg/mL in coating buffer) for 2 h at room temperature. The wells were washed twice with TMS (2x200μL) and blocked with 100 μL TMS with 1% of BSA for 30 min at room temperature. After 1x200μL wash with PBS, the wells were incubated at 37°C with 50 μL DC-SIGN-Fc (3 μg/mL) in TMS with 1% of BSA for 1 h. The wells were washed four times with TMS (4x200μL) and incubated at room temperature with 50 μL of Goat-anti human HRP (0.8 μg/mL) in TMS with 1% of BSA for 30 min. After four washes with TMS (4x200μL), 100 µL of substrate solution (3,3′,5,5′-Tetramethylbenzidine, TMB, in citric/acetate buffer, pH=4, and H_2_O_2_) were added and after 4 min at room temperature the reaction was stopped with 50 µL of H_2_SO_4_ (0.8M) and the plate was read at 450 nm ELISA reader. All the experiments were performed in triplicate.

### Statistical methods

Multiple t-test was used to determine differences between the experiments and the control conditions. *p*-value ≤0.05 is considered to be statistically significant (Graphad Prism 6.00). One asterisk indicates a *p*-value < 0.05; two asterisks indicate a *p*-value < 0.01 and three asterisks indicate a *p*-value < 0.001.

## Results and Discussion

**Figure 1:**
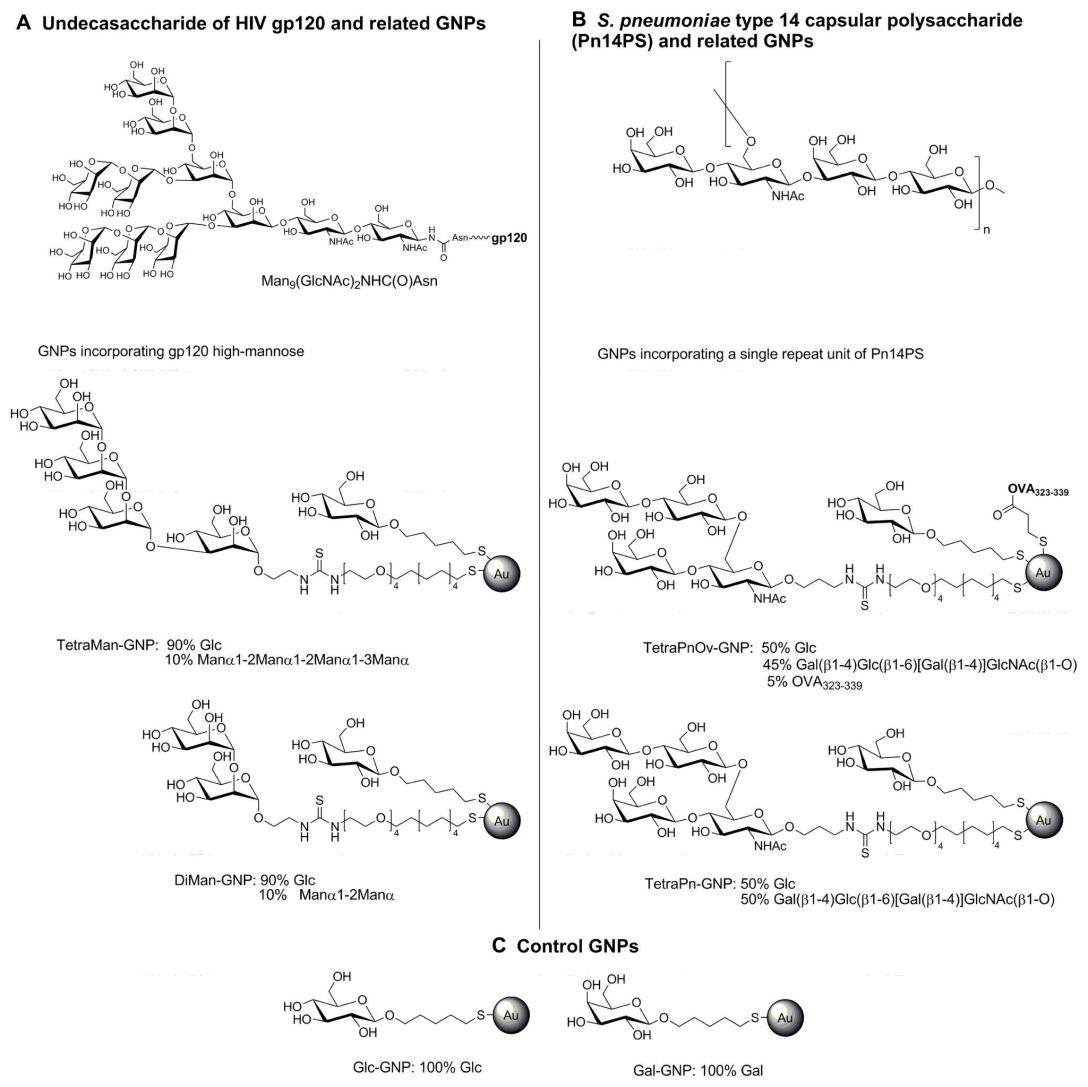
Gold glyconanoparticles used in this work to coat ELISA plates for anti-carbohydrate-antibodies detection. (**A**) High-mannose type undecasaccharide present on the HIV gp120 surface and GNPs carrying the tetramannoside (TetraMan) or dimannoside (DiMan), partial structures of the viral gp120 high-mannose undecasaccharide, to detect 2G12 antibody. (**B**) Repeating unit of *S*. *pneumoniae* type 14 capsular polysaccharide and GNPs carrying the tetrasaccharide epitope (TetraPn) of the *S*. *pneumoniae* Pn14PS and the T-helper OVA_323-339_. (**C**) GNPs carrying glucose or galactose as control.

To validate the GNP-ELISA we have selected as antigens a panel of GNPs (Figure 1) that we have previously prepared to investigate glycan/protein interactions and as a carrier for carbohydrate-based vaccine candidates: Two of them (Figure 1A) carry the disaccharide Man(α1-2) Man(α1→) (DiMan-GNP) or the tetrasaccharide Man(α1-2) Man(α1-2) Man(α1-3) Man(α1→) (TetraMan-GNP) that are present in the high-mannose type glycans of HIV glycoprotein gp120 [18]. Another set (Figure 1B) is formed by GNPs that carry the tetrasaccharide Gal(β1-4) Glc(β1-6) [Gal(β1-4)] GlcNAc(1→) epitope of the *S. pneumoniae* type 14 polysaccharide, alone (TetraPn-GNP) or in combination with the small peptide OVA_323-339_ of ovalbumin (TetraPnOv-GNP) [19]. As a control (Figure 1C), GNPs bearing only glucose (Glc-GNP) or galactose (Gal-GNP) were also included. The oligosaccharides are conjugated to the same aglycon, a thiol-ending amphiphilic linker to attach them to the gold surface. A glucose conjugate is incorporated as inner component to modulate the density of the antigenic oligosaccharides on the surface [22]. Nunc MaxiSorp plates were selected for the GNP-ELISA, as similar modified polystyrene slides were previously used to prepare microarrays of polysaccharides and proteoglycans [23]. GNPs were adsorbed on the MaxiSorp surface due to their high hydrophilicity.

### Detection of anti-HIV monoclonal antibody 2G12

As a proof of principle, we set up a GNP-ELISA for the detection of the anti-HIV human monoclonal antibody 2G12. The 2G12 antibody is one of the broadly neutralizing antibodies against HIV-1 and binds to a conserved high-mannose cluster on HIV gp120 [24]. GNPs carrying selected gp120 high-mannose oligosaccharides were previously shown to bind 2G12 and to compete with 2G12/gp120 binding as demonstrated by surface plasmon resonance (SPR), NMR, and cellular neutralization experiments [25]. In particular, TetraMan-GNPs were able to bind 2G12 with high avidity (nanomolar range) and inhibit 2G12/gp120 interaction in the micromolar range as measured by SPR and NMR. On the contrary, the analogue DiMan-GNPs did not show significant binding to 2G12 even at higher concentration [25]. For this reason, in the present study, we selected TetraMan-GNP for the detection of 2G12 and DiMan-GNP as control to exclude non-specific interactions.

Following the standard procedure for ELISA antigens coating, the wells were coated with a solution of TetraMan-GNP, DiMan-GNP, and Glc-GNP at different concentrations (100, 10, and 1 µg/mL). Glc-GNP was included as a negative control. We observed in our experiments that multiple Tween washes decreased the sensitivity of the detection (data not shown), so we decided to wash the plate with PBS (10 mM, pH 7.4) before blocking with 1% BSA. Next, 2G12 was added at 2.4 µg/mL (16.5 nM) concentration and incubated for 1 h at room temperature followed by detection with horseradish peroxidase (HRP)-conjugated goat anti-human IgG.

**Figure 2:**
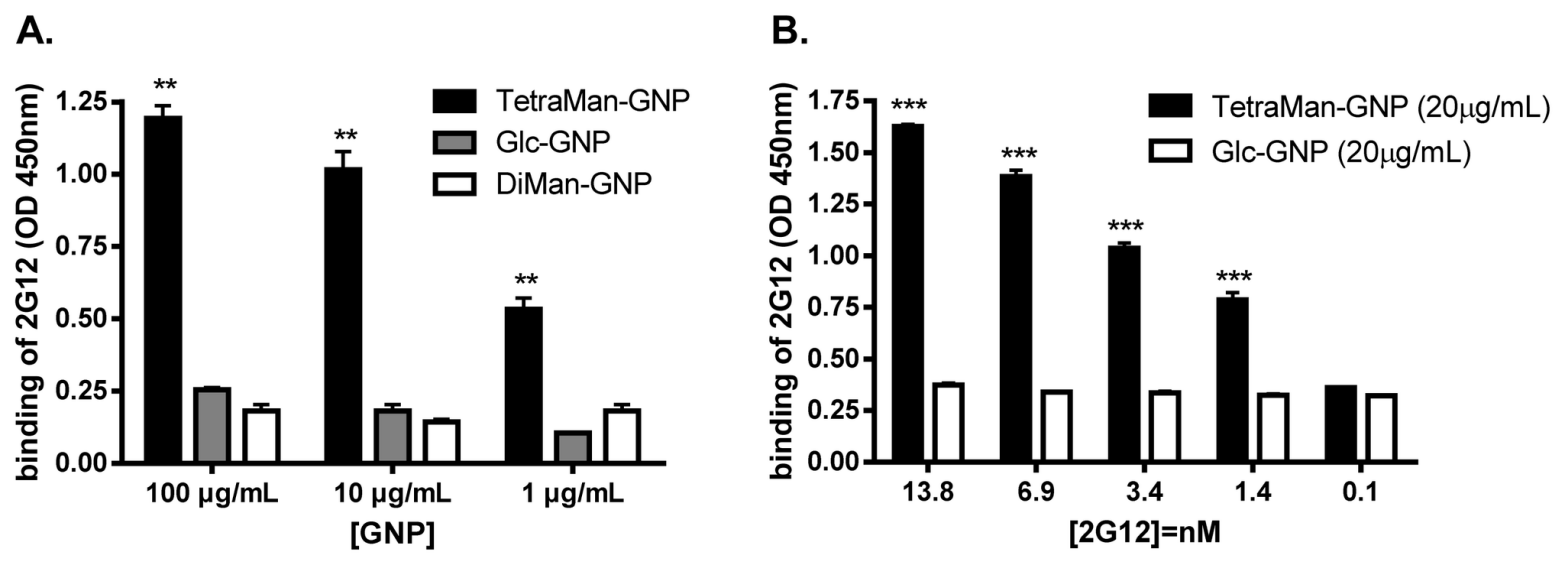
Detection of anti-HIV monoclonal antibody 2G12 by GNP-ELISA. (**A**) TetraMan, DiMan and Glc-GNP at different concentrations (100, 10, and 1 µg/mL) were used for ELISA plates coating. Antibody 2G12 (2.4 µg/mL, 16 nM) recognizes TetraMan-GNP in a coating-concentration dependent manner, while DiMan-GNP and Glc-GNP are not recognized. Differences between TetraMan-GNP and both control GNPs are significant, as indicated with two asterisks (*p*<0.01). (**B**) Limit of 2G12 detection with TetraMan-GNP: 20 µg/mL of GNPs were used to coat the plate and subsequently incubated with different concentrations of 2G12 (13.8 to 0.1 nM). Error bars represent the standard deviation of three different experiments. Differences between TetraMan-GNP and both control GNPs are significant, as indicated with three asterisks (*p*<0.001). Background signal due to non-specific interactions was around OD 0.25.

Figure 2A shows the concentration-dependent response of 2G12 towards the GNPs measuring the optical density (OD) at 450 nm. Even at 1 µg/mL of coating, TetraMan-GNP was able to induce a significant signal (OD ~ 0.5) after incubation with 2G12. However, 2G12 did not interact with the DiMan-GNP at the tested concentrations. The negative response of the DiMan-GNP excluded non-specific interactions (due to the linker or gold) between 2G12 and the gold nanoparticles.

Glc-GNP was not recognized by the antibody (Figure 2) excluding also non-specific interactions because both TetraMan-GNP and DiMan-GNP have 90% of Glc conjugate on their surface. 2G12 recognized neither GNPs coated with OVA_323-339_ nor GNPs bearing a branched high-mannose pentasaccharide (Figure S1 in File S1; for the structures of PentaMan-GNP and OVA/Glc-GNP, see Figure S6 in File S1).

When the monomeric TetraMan oligosaccharide conjugated to 2-aminoethyl linker was employed to coat the Nunc MaxiSorp plate under the same conditions as those for GNPs coating, no 2G12 response was detected (data not shown). GNPs with increasing density of TetraMan (50%) led to a more sensitive 2G12 detection in comparison to the 10% TetraMan-GNP at the same concentration (25 µg/mL) (Figure S2 in File S1). The 50% TetraMan-GNP contains 3 times more mannosides at the same mg/mL concentration (Text S1 in File S2).

These results suggest that the multivalent presentation of the oligomannosides on the gold nanoparticles, used as antigens in the ELISA coating, provides high selectivity and sensitivity for the detection of 2G12.

To get a deeper insight on the sensitivity of the GNP-ELISA, 20 µg/mL of GNPs were used to coat the ELISA plates and different concentrations of 2G12 (from 13.8 nM to 0.1nM) were subjected to analysis (Figure 2B). The GNP-ELISA with TetraMan-GNP allowed the detection of 2G12 at 1.4 nM (0.2 µg/mL). Considering that the concentration of 2G12 in plasma (in animal models) ranges between 1200 to 49 µg/mL [26], our results indicated that GNP-ELISA is a valid method for the detection of very low levels of anti-carbohydrate antibodies that could be also applied for biological samples. Our GNPs add a new multivalent tool to the described glycan arrays of covalently coupled oligomannose dendrons [9] and to the virus capsides oligomannoside conjugates used in ELISA as antigens [27] for 2G12 detection. The detection limit in the glycans array with the dendrons [9] or in the conventional ELISA plates with the virus conjugates [27] is 0.05 and 0.5 μg/mL, respectively, while the GNP-ELISA is able to detect 0.2μg/mL of 2G12.

### GNP-ELISA for the detection of anti-carbohydrates antibodies in mice

**Figure 3:**
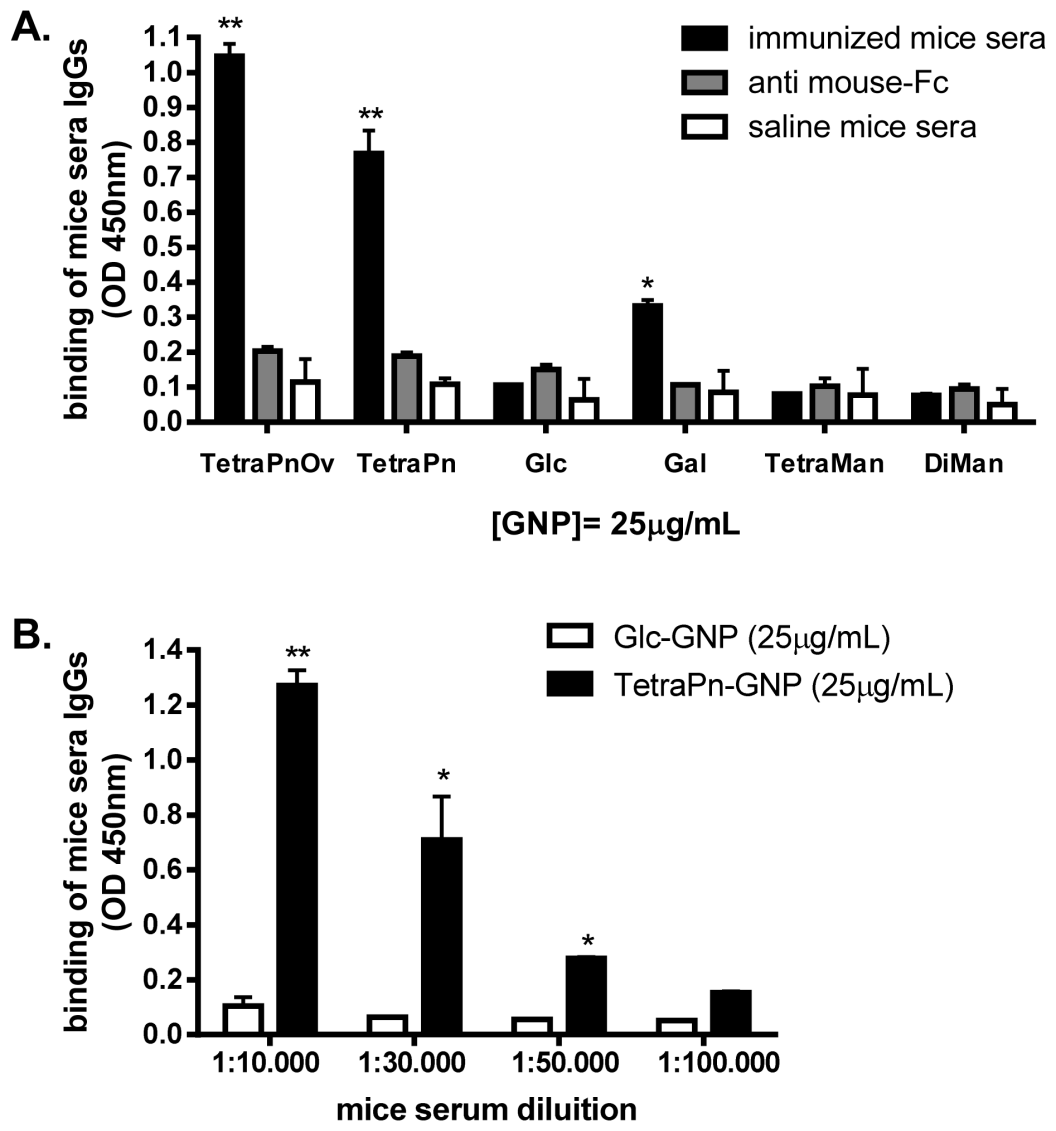
GNP-ELISA for the detection of anti-carbohydrates antibodies from mice immunized with TetraPnOv-GNP. (**A**) Detection of specific IgG by GNPs carrying different carbohydrates. TetraPnOv- and TetraPn-GNPs show strong binding to mice serum at a 1:30,000 dilution. Detectable binding was also observed for Gal-GNP. Glc-, TetraMan-, and DiMan-GNPs were not recognized by the sera’s IgG. Non-specific interactions of the secondary anti-mouse IgG with the GNPs were excluded performing the GNP-ELISA in the absence of 2G12. Sera of mice immunized with saline were used as negative control. Differences between sera from immunized mice and control samples are significant, as indicated with one (*p*<0.05) or two asterisks (*p*<0.01); (**B**) ELISA plate coated with 25 µg/mL of TetraPn-GNP carrying *S*. *pneumoniae* or Glc-GNP (control) were used to determine the detection limit for anti-TetraPn antibodies in mice sera. GNP-ELISA was able to detect antibodies up to 1:50,000 dilutions of sera. Differences between TetraMan-GNP and Glc- GNP are significant, as indicated with one (*p*<0.05) or two asterisks (*p*<0.01).

The next step was to verify the method for the detection of anti-carbohydrate IgG antibodies in a more complex biological sample. We have previously demonstrated that TetraPnOv-GNP bearing a 40% of the synthetic epitope TetraPn, which corresponds to the single repeating unit of the *S. pneumoniae* type 14 capsular polysaccharide (Pn14PS) [28], and a 5% of the T-cell epitope OVA_323-339_ (Figure 1) are able to evoke functional anti-carbohydrate IgG antibodies in mice against Pn14PS [19]. In that work, the detection of the specific IgGs was performed by coupling the tetrasaccharide epitope (Tetra-Pn) to BSA and running a “classic” ELISA for IgG antibodies diluting mice sera from 1:10 to 1:1000. Here, TetraPnOv-GNP (25 µg/mL) was directly used to coat ELISA plates for the IgG detection (Figure 3). TetraPn-GNP carrying 50% of tetrasaccharide and 50% glucose was also tested in the ELISA in order to exclude sera interactions with the OVA_323-339_ peptide. TetraMan-GNP, DiMan-GNP, Glc-GNP, and Gal-GNP were used as control. Sera from mice immunized with TetraPnOv-GNP were diluted 1:30,000. Specific IgGs against TetraPn recognized TetraPnOv- and TetraPn-GNPs on the ELISA plate with high OD at 450 nm (Figure 3A). High levels of IgGs were detected in serum of mice immunized with the TetraPnOv-GNP (OD > 1) and significant signal (~0.8 OD) was also detected for TetraPn-GNP. GNPs bearing 5% of OVA peptide and 95% of glucose were not detected by mice sera IgGs (Figure S3 in File S1).

No response was detected after coating the ELISA plate with Glc-GNP, indicating the absence of significant titers of anti-glucose antibodies in the serum of mice immunized with the TetraPnOv-GNP (that contains 50% of glucose). A weak positive but significant signal (OD > 0.2) was observed for Gal-GNP, in agreement with the molecular structure of the biantennary TetraPn (Figure 1) that presents a terminal galactose in both antennas. As expected, serum antibodies showed no affinity for mannosides, as both TetraMan- and DiMan-GNPs were not able to capture any component of sera from mice immunized with TetraPnOv-GNP. The presence of specific IgGs against TetraPn was also detected in mice immunized with Pn14PS conjugated to cross reactive material from diphtheria toxin (Pn14PS-CRM) [19] (Figure S4 in File S1). Sera from mice immunized with saline were used as negative control and gave no signal (OD <0.2) in the GNP-ELISA (Figure 3A). The secondary anti-mouse IgG antibody did not react with any of the GNPs, so that non-specific interactions were excluded.

**Figure 4:**
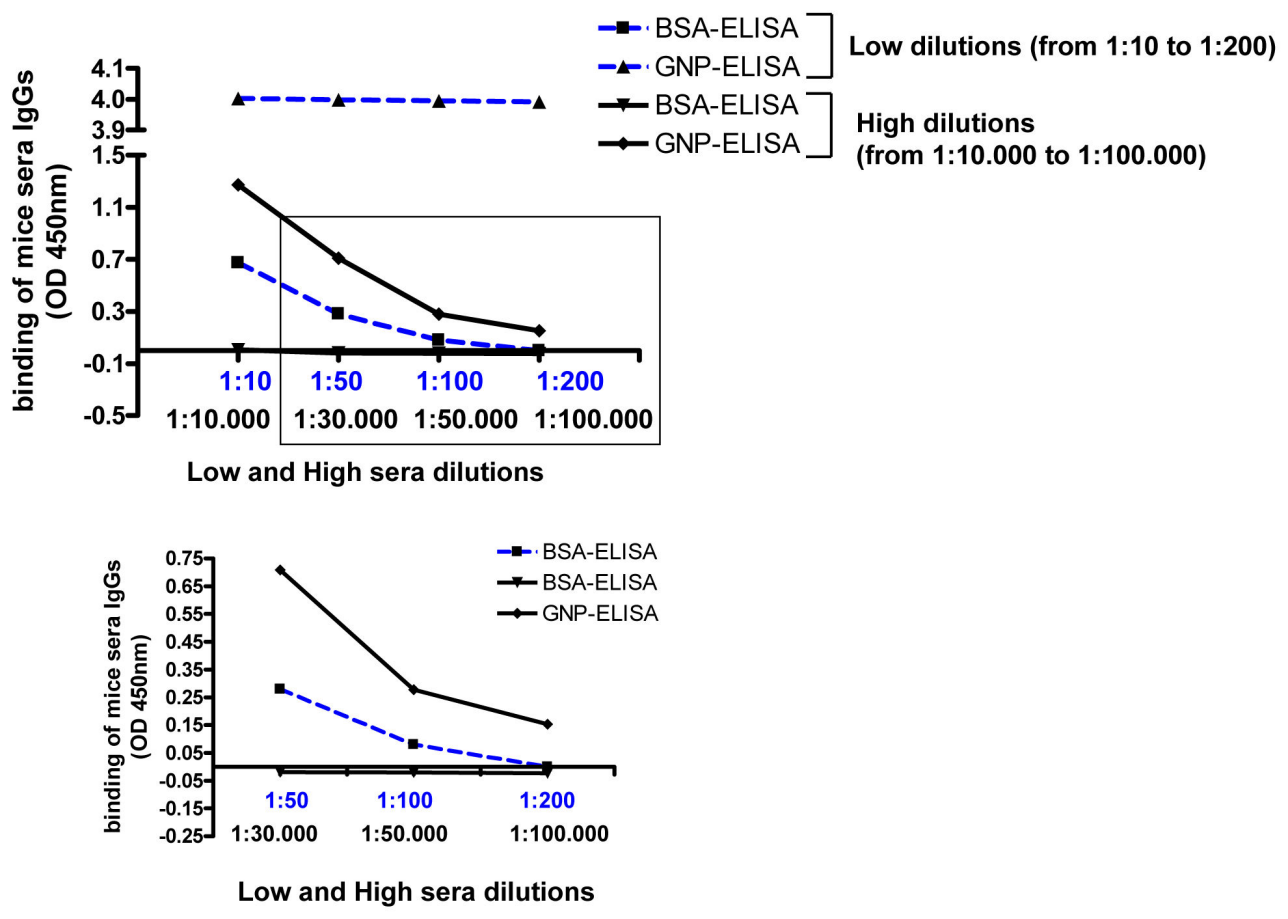
Direct comparison between BSA-ELISA and GNP-ELISA for IgGs detection in mice. At low sera dilution (dotted blue lines, from 1:10 to 1:200) the BSA-ELISA (blue line, squares) gave a response from 0.7 to 0.1 OD, while the GNP-ELISA (blue line, triangle) gave a saturated response around 4 OD. At high sera dilution (dark lines, from 1:10.000 to 1:100.000) the BSA-ELISA (dark line, reverse triangle) was no able to detect IgGs (OD around zero), while the GNP-ELISA (dark line, rhombus) detected IgGs (OD from 1.4 to 0.3) up to 1:50.000 diluted sera.

Serial dilutions (1:10,000 to 1:100,000) of sera from mice immunized with TetraPnOv-GNP were analyzed on plates coated with 25 µg/mL of TetraPn-GNP (Figure 3B). The OD at 450 nm coming from the specific anti-carbohydrate IgGs binding was detected up to 1:50,000 dilution. In comparison to the classical ELISA used in our previous work [19] a ~3,000-fold increase of detection was achieved by this new method based on GNPs (Figure 4, and Figure S5 in File S1). The classical ELISA for the detection of anti-carbohydrate was able to detect IgGs in mice sera from l: 10 to 1:50 sera dilution, with OD ~ 0.65 at 1:10 dilution. Using the GNP-ELISA methodology we were able to detect specific anti-carbohydrate IgGs from 1:10.000 to 1:50.000 sera dilution, with an OD ~ 0.65 at 1:30.000 dilution (Figure 4 and Figure S5 in File S1). These results demonstrate that the GNP-ELISA represents a novel, straightforward screening method for detecting anti-carbohydrate antibodies evoked by carbohydrate-based vaccines.

The estimated total protein concentration in the immunized mice sera was ~20mg/mL as determined by UV absorbance at 280nm [29]. Purification of mice sera by Protein G-Sepharose high performance columns, led to ~0.2 mg/mL of total IgGs in the mice sera (Text S2 in File S2). From these results and from the dilution we used in our work, ng/mL of anti-carbohydrate antibodies were detected while with the classic approach (BSA-ELISA) only μg/mL of antibodies were detected.

The method could be extended to screen the affinity of any carbohydrate, once tailored onto the multivalent GNP nanoplatform. The GNP-technique is more sensitive than ELISA based on glycoconjugation to proteins, probably due to the higher glycan density on the 3D surface of the gold nanoclusters (Figure S5 in File S1). This new approach also affords an easier and faster procedure to monitor the antibody titers during animal immunization studies as the same GNP construct can be used both for the immunization and for screening the antibody titers in ELISA. The GNP-ELISA approach may also allow multiple screening of complex samples for the detection of anti-carbohydrate antibodies by using GNPs displaying different carbohydrate antigens.

### Dendritic cells adhesion assay using GNP-ELISA

**Figure 5:**
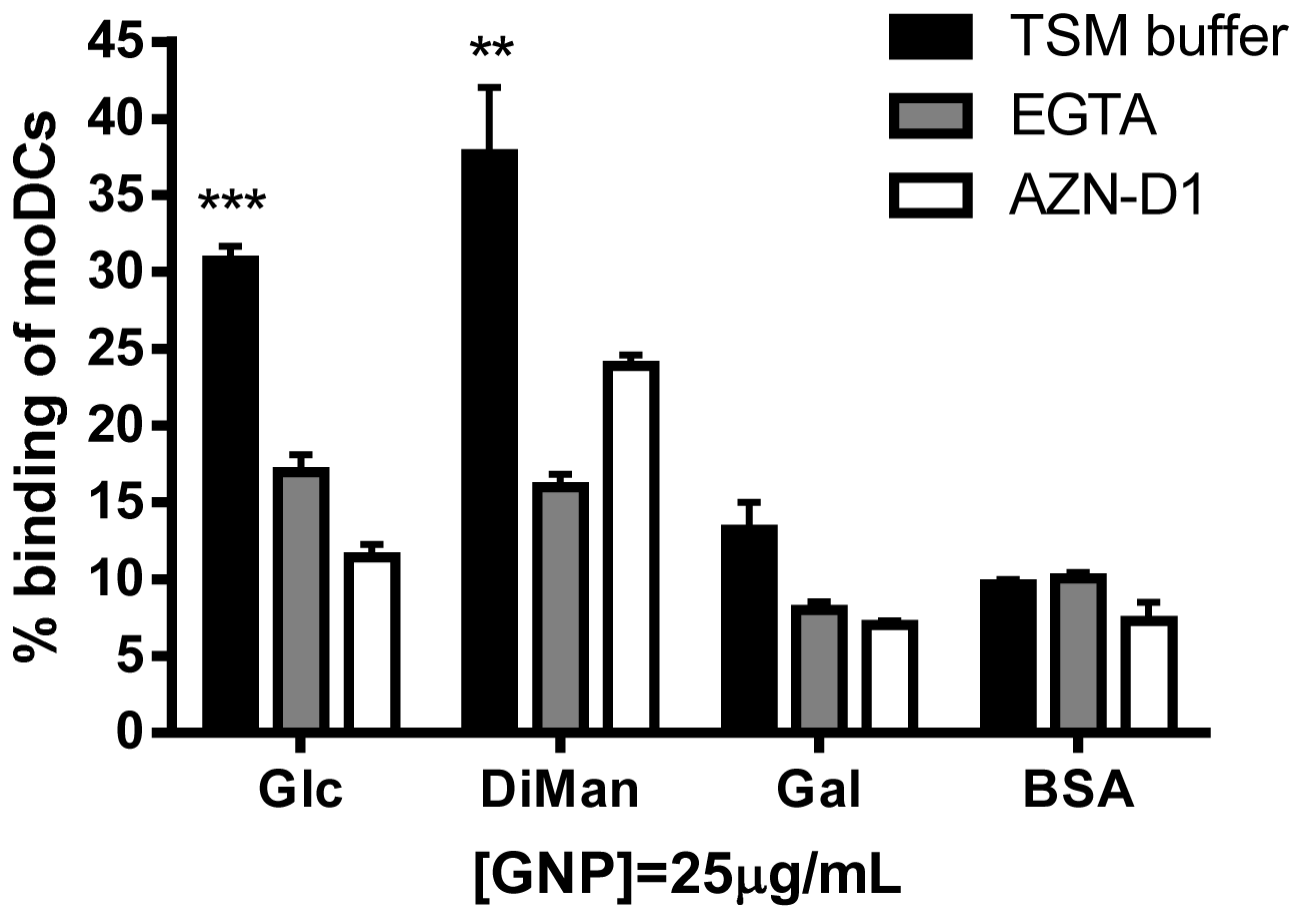
Dendritic cells adhesion assay using GNP-ELISA. Dendritic cells show different carbohydrate-affinity. Binding of moDC to GNPs in calcium and magnesium containing buffer, was determined using plate adhesion assay in the presence or absence of EGTA (3.75 nM) or anti-DC-SIGN antibody AZN-D1 (10 μg/mL). These experiments were performed at least four times with similar results. Each experiment was performed in triplicate. Error bars indicate standard deviations. The binding to the GNPs was significantly decreased when treated with EGTA or AZN-D1, as indicated with one (*p*<0.05) or two asterisks (*p*<0.01).

The application of the multivalent GNP-coated surface was extended to an adhesion assay with human dendritic cells (DC) to evaluate the potentiality of this new ELISA coating for studying cellular carbohydrate-mediated interactions. DCs are antigen-presenting cells that display calcium-dependent glycan-binding proteins (C-type lectins) on their surface, which function in the recognition and internalization of pathogens [30]. One of the C-type lectin expressed in DC is DC-SIGN (Dendritic Cell Specific Intracellular Adhesion Molecule Grabbing Non-integrin) with dual specificity for mannose- and fucose-containing glycans [31,32]. To evaluate binding of DC to different GNPs, ELISA wells were coated with Glc-GNP, DiMan-GNP, and Gal-GNP. It has been previously shown that DiMan-GNP inhibits gp120(CN54) binding to DC-SIGN in the nanomolar range [18]. Binding of moDC was determined using a calcium and magnesium containing buffer in the absence and presence of ethylene glycol tetra acetic acid (EGTA) or the anti DC-SIGN blocking mAbs AZN-D1 [33]. This antibody was previously shown to block the DC internalization of fluorescent-GNPs carrying oligomannosides [34]. The GNP-modified plate was incubated with calcein-labeled monocyte-derived DC (moDC) following a protocol commonly used to investigate the binding of moDC with glycoconjugates [21]. The moDC showed a specific carbohydrate-dependent binding to the selected GNPs, as shown in Figure 5. DiMan-GNP exhibited the highest affinity for moDC (around 40% binding), while a weak binding (~15%) was detected for Gal-GNP. This low binding is in agreement with the reported low adhesion of DC to PAA-coupled galactose [21]. The significant signal detected for Glc-GNP (30%) can be explained by the previous evidence that glucose at high concentrations inhibits the binding of high-mannose glycoproteins to DC-SIGN [35].

The presence of EGTA 3.75 mM blocked the binding of the moDC to both DiMan-GNP and Glc-GNP, indicating the involvement of calcium-dependent C-type lectins (Figure 5, grey bars). Pre-treatment of moDC with AZN-D1 (10 μg/mL) significantly decreases their binding to Glc-GNP and, in a less extent, to DiMan-GNP. This result suggests that DC-SIGN is involved in the binding of moDC to DiMan and Glc-GNPs.

### DC-SIGN binding to GNPs

**Figure 6:**
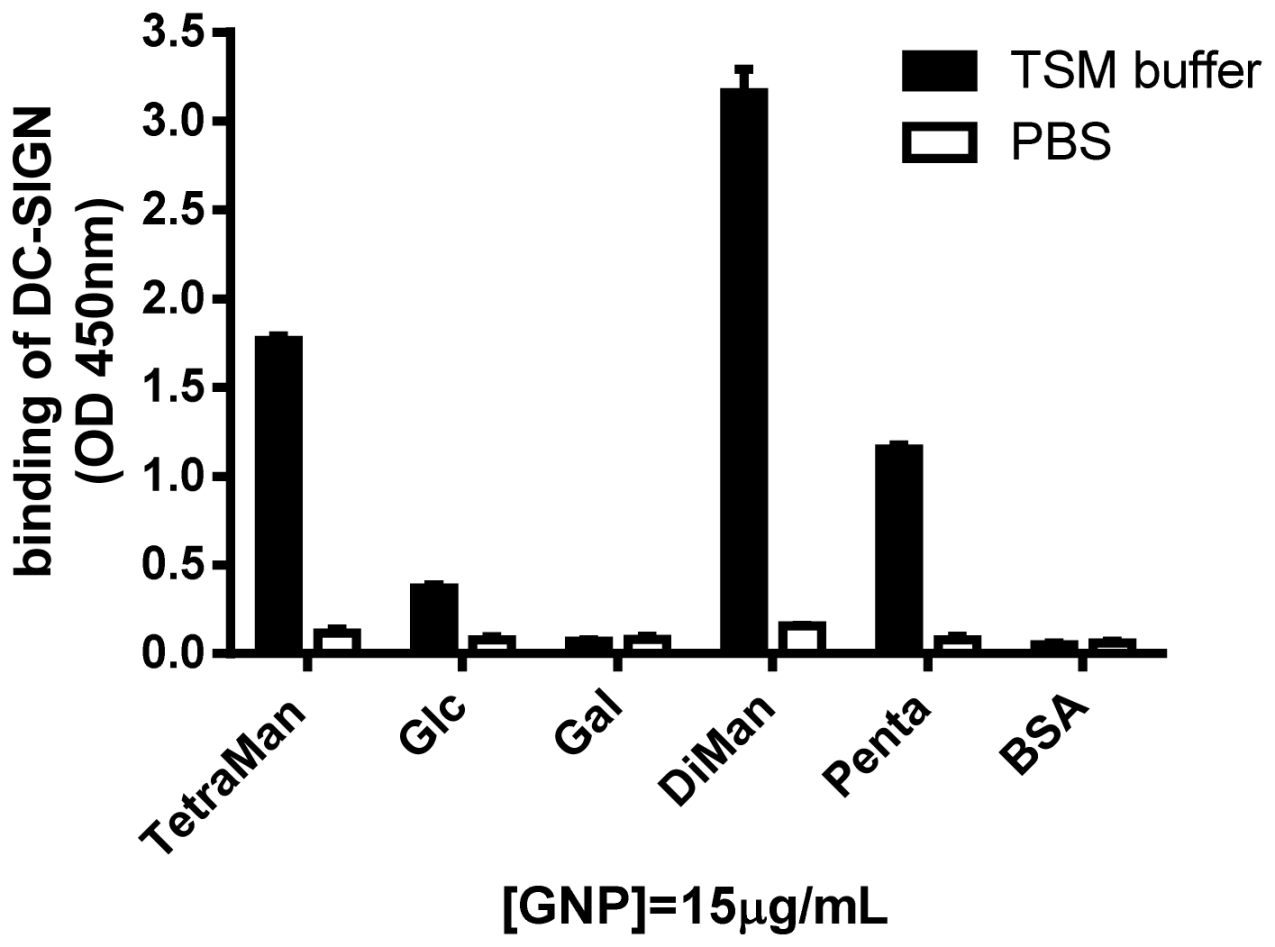
DC-SIGN-Fc binding to GNPs. Binding was determined using GNPs-ELISA in PBS and in calcium and magnesium containing buffer (TMS). These experiments were performed in triplicate at least three times with similar results. Error bars indicate standard deviations.

To extend the GNP-ELISA to other carbohydrate-binding proteins, the binding of a recombinant chimera protein DC-SIGN-Fc to different sugar-coated GNPs was tested (Figure 6). DC-SIGN-Fc was produced in Chinese hamster ovary K1 cells by co-transfection of DC-SIGN-Sig-pIgG1 Fc (20 μg) and pEE14 (5 μg) vector. DC-SIGN-Fc consists of the extracellular portion of DC-SIGN (residues 64 to 404) fused at the C-terminus to a human IgG1/Fc fragment into the Sig-pIgG1-Fc vector [36]. DC-SIGN-Fc bound to TetraMan-, DiMan- and PentaMan-GNPs in the presence of calcium and magnesium containing buffer (TMS). The binding of DC-SIGN to DiMan-GNP, TetraMan and PentaMan-GNPs was very high (OD>1) in agreement with the carbohydrate-specificity of this lectin [18,32]. Glc-GNP exhibited significant lower binding (OD~0.3) than the mannoside GNPs, while no binding was detected for Gal-GNP (OD<0.1) and BSA. No binding was detected in PBS. This result indicates that DC-SIGN is involved in the adhesion of DC to the carbohydrate-modified plate and validate the GNP-ELISA for lectin detection and interactions. The selective adhesion of DCs and recombinant lectin to GNPs confirms also the coating of the ELISA surface with the multivalent GNPs and indicates that GNP-ELISA can be used in solid-phase assays to explore glycan-binding properties of lectins as well as whole cells. The binding of bacteria [37–39] and mammalian cells [40] to carbohydrates have been also probed in microarray systems and our new ELISA approach can contribute to cellular studies on solid-phase.

## Conclusions

A new fast and sensitive method (GNP-ELISA) for the detection of anti-carbohydrates antibodies and other glycan-binding proteins has been developed by using multivalent and high molecular weight GNPs as solid-phase coating. GNPs are three-dimensional systems that allow a high valence in the presentation of selected glycans (up to 100 copies per nanoparticle) on a 2 nm gold nanoclusters. This high concentration of glycans in a small surface could be responsible for the high sensitivity of our assay. The possibility of varying density and type of carbohydrate antigen on the nanoparticles [41] makes the GNP-ELISA a versatile and sensitivity method for multiplex detection of carbohydrate-binding partner comparable to printed glycan-array. We first showed the selectivity of GNP-ELISA for detecting the interaction between a tetramannoside of the high-type mannose glycan expressed on HIV glycoprotein gp120 and the human antibody 2G12 at the nM range. Furthermore, we have successfully used GNP-ELISA to highly improve the detection (~3,000-fold) of specific IgGs against *S. pneumoniae* in mice sera respect to the BSA-based ELISA. Finally, we showed that the GNP-ELISA can be used in solid phase cellular binding assays, as demonstrated by the selective binding of human moDC and the lectin DC-SIGN on the multivalent surface. Gold nanoparticles have previously been used in immunosensing by profiting of the unique physical properties of metallic nanoclusters [42]. The simplicity, the high sensitivity and the versatility of the GNP-ELISA method, represents a new approach to basic studies of protein-carbohydrate interactions that can be especially useful for vaccination studies and clinical identification of biomarkers.

## Supporting Information

File S1 **Figures related to different control experiments (Figures S1-S6) described in the manuscript are available in the File S1.** A comparison between the GNP-ELISA and the BSA-ELISA is reported in Figure S5 in File S1.
(DOC)

File S2 **Difference in the tetramannoside loading between the 50% and the 10% TetraMan-GNPs (Text S1 in File S2) and details of purification of IgGs from mice sera (Text S2 in File S2) are also available.**
(DOC)
