# Supplementary material for: High Sensitive Detection of Carbohydrate Binding Proteins in an ELISA-Solid Phase Assay Based on Multivalent Glyconanoparticles
Source: PLoS One. 2013 Aug 27;8(8):e73027. doi: 10.1371/journal.pone.0073027 (PMC3754922; doi:10.1371/journal.pone.0073027)
Supplement: File S1 — Figures related to different control experiments (Figures S1-S6) described in the manuscript are available in the File S1. A comparison between the GNP-ELISA and the BSA-ELISA is reported in Figure S5 in File S1. (DOC) [file pone.0073027.s001.doc]

**SUPPORTING FILE S1 for**

High Sensitive Detection of Carbohydrate Binding Proteins using an ELISA-Solid Phase Assay based on Multivalent Glyconanoparticles

Fabrizio Chiodo, Marco Marradi, Boris Tefsen, Harm Snippe, Irma van Die, and Soledad Penadés

**Figure S1**: Detection of anti-HIV monoclonal antibody 2G12 (1 μg/mL) with 25 µg/mL of different GNPs. TetraMan-GNP are able to detect 2G12 at these concentrations. PentaMan-GNP, carrying 90% of glucose and 10% of pentamannoside, and OVA/Glc-GNP, carrying 5% of OVA peptide and 95% of glucose, do not recognize 2G12. These GNPs behave similar to DiMan-GNP and Glc-GNP.

**Figure S2**: Comparison of the detection of 2G12 by GNPs carrying 10% or 50% of TetraMan. The 50% TetraMan-GNPs led to a more sensitive 2G12 detection in comparison to the 10 % TetraMan-GNPs. No detection was observed for Glc-GNPs.

**Figure S3**: Detection of specific anti-carbohydrates IgG antibodies from mice immunized with TetraPnOv-GNP by ELISA plate coated with GNPs carrying different carbohydrates. TetraPnOv-GNP and TetraPn-GNP show strong binding to mice serum at a 1:30,000 dilution. OVA/Glc-GNP (carrying 5% of OVA_323-339_ and 95% of glucose) and Glc-GNP (coated only with glucose) were not recognized by the sera’s IgG. Sera of mice immunized with saline were used as negative control.

**Figure S4**: GNP-ELISA for the detection of specific anti-carbohydrates IgG antibodies from mice immunized with Pn14PS conjugated to cross reactive material from diphtheria toxin (Pn14PS-CRM-197 conjugate) and from mice immunized with TetraPnOv-GNP. Sera were diluted 1:30,000. Specific IgGs recognized TetraPnOv- and TetraPn-GNPs on the ELISA plate with high OD at 450 nm. Higher IgGs levels were detected in serum of mice immunized with the Pn14PS-CRM than in serum of mice immunized with TetraPnOv-GNP.


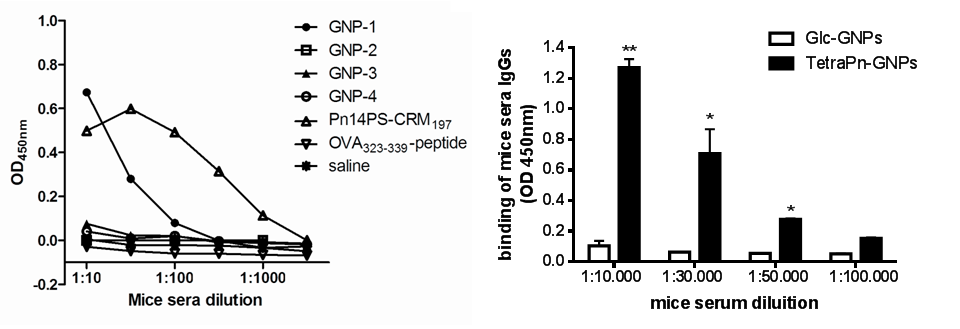


**Figure S5**: Comparison between BSA-TetraPn conjugate and TetraPn-GNP as antigens for ELISA in the detection, under the same conditions, of IgG from sera of the immunized mice. *Left*: Detection of IgG against BSA-TetraPn used as antigen to coat ELISA plates. Mice immunized with Pn14PS-CRM and mice immunized with TetraPnOv-GNP (GNP-1) showed high amount of specific IgG at 1:10 to 1:100 dilution (see reference 19 in the main article). *Right*: Detection of IgG from mice immunized with TetraPn-GNPs and used as antigen to coat ELISA plates. Specific IgGs were detected up to 1:50,000 dilution. The comparison clearly shows a ~3000 fold improvement in IgGs detection. BSA-TetraPn conjugate has a tetrasaccharide/protein ratio of approximately 5, while the average number of tetrasaccharide units per TetraPn-GNP is 36.


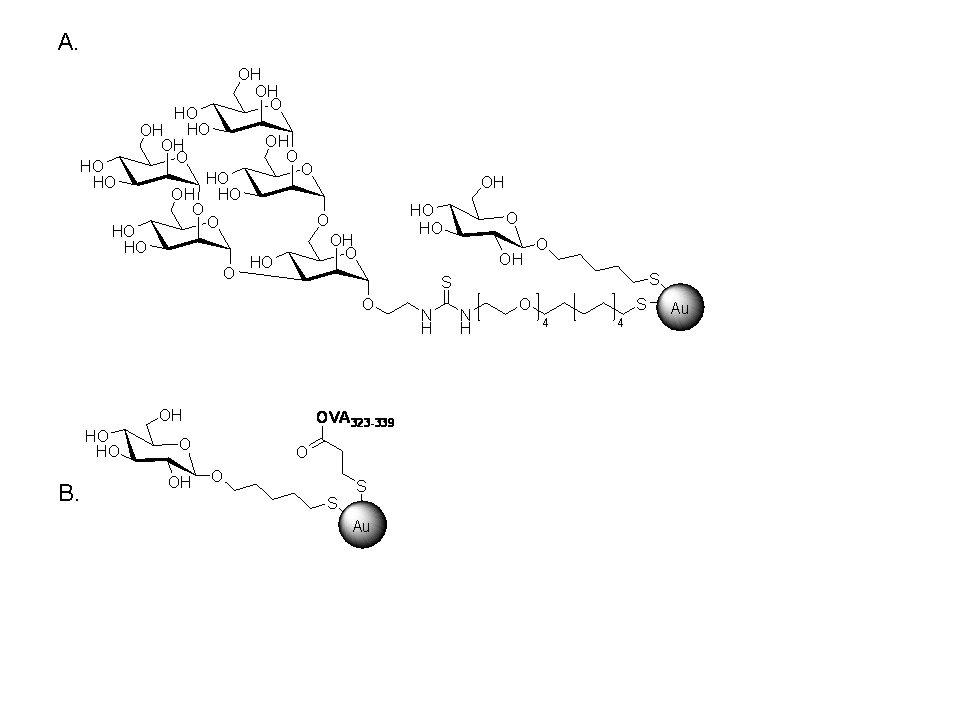


**Figure S6**: Other GNPs used in this study: A) PentaMan-GNP: GNP carrying 90% glucose and 10% of the pentamannoside Man(α1-2)Man(α1-3)[Man(α1-2)Man(α1-6)]Man(α1→) (PentaMan); B) OVA/Glc-GNP: GNP carrying 5% of OVA_323-339_ and 95% of glucose.
