# Supplementary material for: High Sensitive Detection of Carbohydrate Binding Proteins in an ELISA-Solid Phase Assay Based on Multivalent Glyconanoparticles
Source: PLoS One. 2013 Aug 27;8(8):e73027. doi: 10.1371/journal.pone.0073027 (PMC3754922; doi:10.1371/journal.pone.0073027)
Supplement: File S2 — Difference in the tetramannoside loading between the 50% and the 10% TetraMan-GNPs (Text S1 in File S2) and details of purification of IgGs from mice sera (Text S2 in File S2) are also available. (DOC) [file pone.0073027.s002.doc]

**SUPPORTING FILE S2 for**

High Sensitive Detection of Carbohydrate Binding Proteins using an ELISA-Solid Phase Assay based on Multivalent Glyconanoparticles

Fabrizio Chiodo, Marco Marradi, Boris Tefsen, Harm Snippe, Irma van Die, and Soledad Penadés

**Text S1. GNPs bearing 10% of TetraMan oligosaccharide *versus* GNPs bearing 50% of TetraMan oligosaccharide.**

The GNP carrying 50% of the TetraMan and 50% of 5-(mercapto)pentyl β-D-glucopyranoside has an average diameter of 1.9±0.5 nm, which corresponds to around 225 gold atoms and an average MW of 123 kDa as calculated from the estimated molecular formula (C_46_H_85_N_2_O_25_S_2_)_56_(C_11_H_21_O_6_S)_56_Au_225_. That means 56 TetraMan molecules/per GNP. The GNP carrying 10% of the TetraMan and 90% of 5-(mercapto)pentyl β-D-glucopyranoside has an average diameter of 1.4±0.7 nm (116 gold atoms) and an average MW of 47 kDa, (C_46_H_85_N_2_O_25_S_2_)_7_(C_11_H_21_O_6_S)_59_Au_116_. The different number of gold atoms in the GNPs results in a different loading of carbohydrate molecules on the gold surface. Thus, 25 µg/mL solution of 10% TetraMan-GNP (47 KDa) correspond to [GNP] = 0.00053 µmol/mL and [TetraMan] = 0.0037 µmol/mL, while 25 µg/mL solution of 50% TetraMan-GNP (123 KDa), correspond to [GNP] = 0.000203 µmol/mL and [TetraMan] = 0.0116 µmol/mL (3 times μmol than 10% GNP).

**Text S2. IgGs purification from mice sera.**

100 µL of serum from mice immunized with TetraPnOv-GNP were diluted with 900 µL of 20 mM sodium phosphate pH 7 and applied to a ProteinG-Sepharose high performance column (HiTrap Protein G HP, GE Healthcare Life Sciences). The purification of IgGs was performed following the manufacturer instructions (Handbook 18-1037-46 AD, Appendix 4, page 142). After elution, the IgGs-containing fractions were detected by measuring the absorbance at 280 nm with a Beckmann Coulter DU800 spectrophotometer. After purification, the recovered IgGs were able to recognize TetraPn-GNP used as coating in a GNP-ELISA experiment.
